# Supplementary material for: LRP-1 Matricellular Receptor Involvement in Triple Negative Breast Cancer Tumor Angiogenesis
Source: Biomedicines. 2021 Oct 9;9(10):1430. doi: 10.3390/biomedicines9101430 (PMC8533426; doi:10.3390/biomedicines9101430)
Supplement: Supplementary file 1 [file biomedicines-09-01430-s001.zip › biomedicines-1354979-SI.pdf]

biomedicines

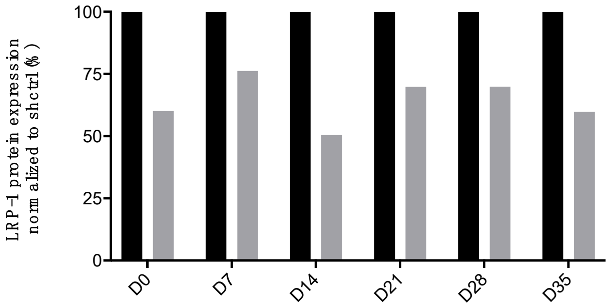

Figure S1. LRP-1 targeted shRNA stability over time. Densitometric analysis of LRP-1 immunoblot in shLRP-1 MDA-MB-231 cells normalized to shCtrl over time without GeneticinTM (G418 Sulfate) selection pressure (n=1).

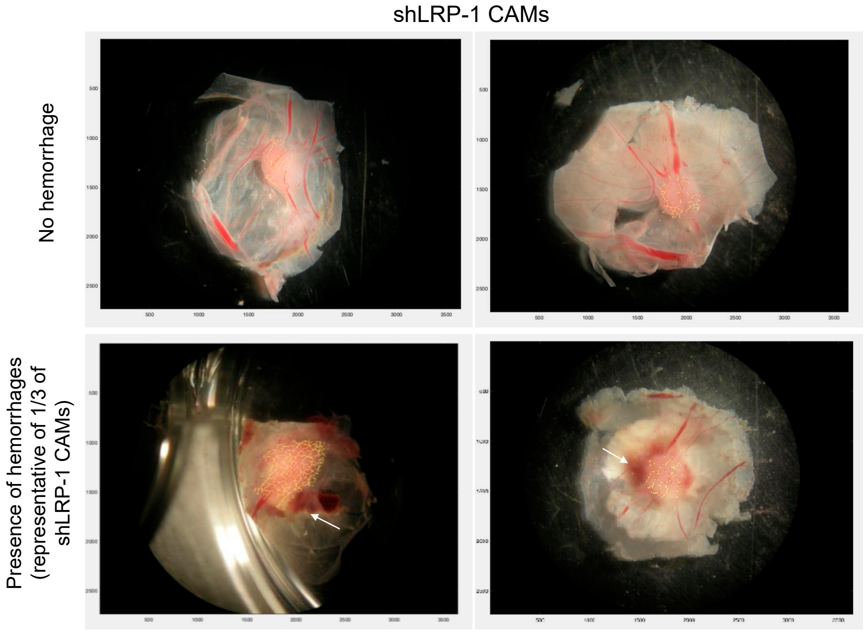

Figure S2. Hemorrhages in shLRP-1 CAMs. (upper panels) Representative macrophotographs of shLRP-1 CAMs without bleeding. (bottom panels) Representative hemorrhage profiles of 1/3 of shLRP-1 CAMs. The bleeding is located by an arrow.

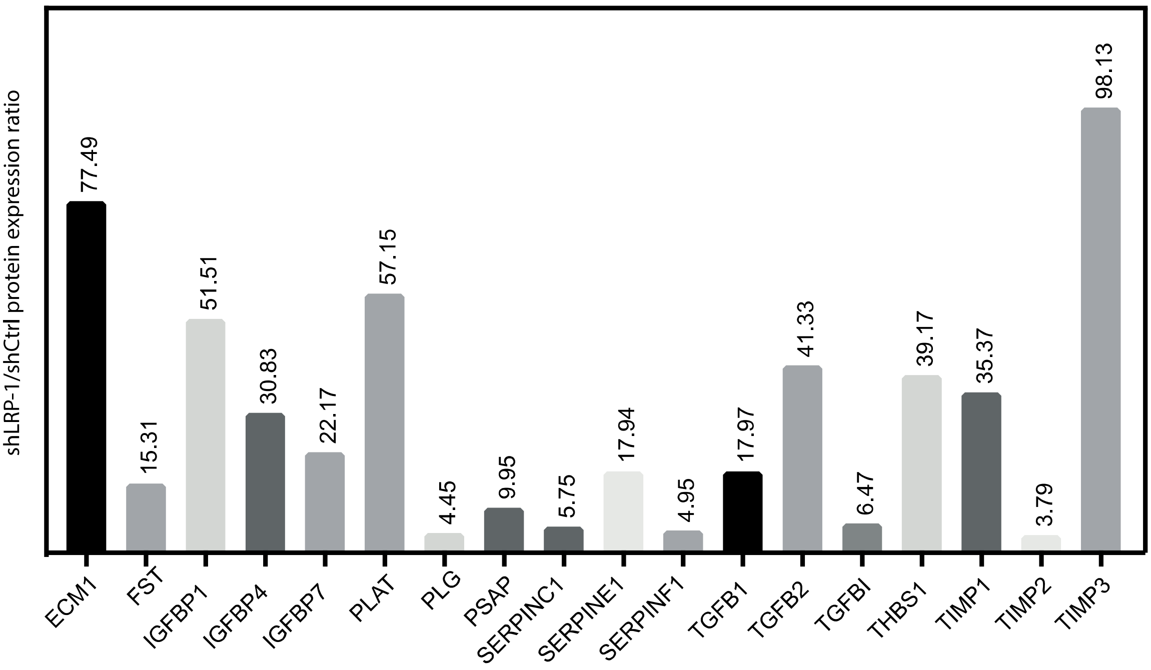

Figure S3. Proteomic enrichment in shLRP-1 secretome. shLRP-1/shCtrl ratio of proteins of interest from proteomics analysis with Proline. All published ratios are significant (p≤0.05). ECM1= Extracellular Matrix Protein 1 ; FST= Follistatin; IGFBP1= Insulin-like growth factor-binding protein 1 ; IGFBP4= Insulin Like Growth Factor Binding Protein 4 ; IGFBP7= Insulin-like growth factor-binding protein 7 ; PLAT= Plasminogen Activator Tissue Type ; PLG= Plasminogen ; PSAP= Prosaposin ; SERPINC1= Serpin Family C Member 1 or Antithrombin ; SERPINE1= Serpin Family E Member 1 or PAI-1 (plasminogen activator inhibitor type I) ; SERPINF1= Serpin Family F Member 1 or PEDF (Pigment epithelium-derived factor); TGFB1= Transforming growth factor beta 1 ; TGFB2= Transforming growth factor beta-2 ; TGFBI= Transforming growth factor beta-induced ; THBS1= Thrombospondin 1 ; TIMP1= TIMP Metallopeptidase Inhibitor 1 ; TIMP2= TIMP Metallopeptidase Inhibitor 2 ; TIMP3= TIMP Metallopeptidase Inhibitor 3.

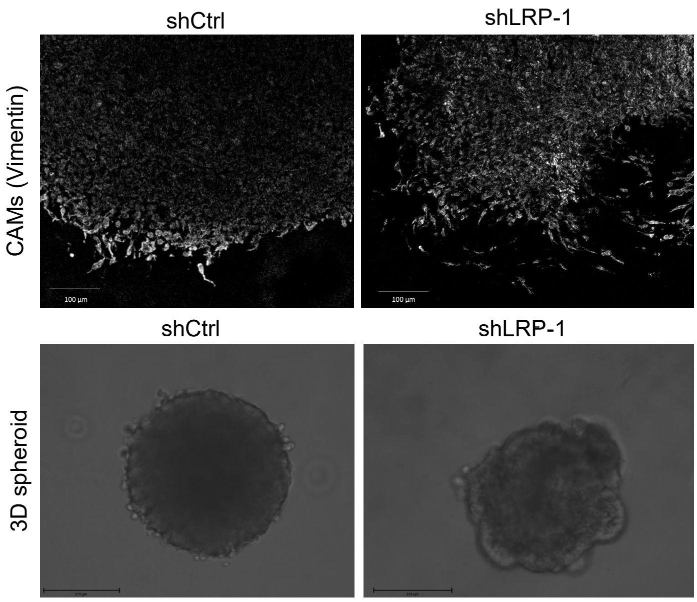

Figure S4. Morphological profile of shLRP-1 CAMs and 3D spheroid. (top panels) Representative microphotography of vimentin immunofluorescence staining in shCtrl and shLRP-1 CAMs. Scale bar: 100µm. (bottom panels) Representative microphotographs of 3D shCtrl and shLRP-1 spheroid 48h after formation. Scale bar: 275µm.

Table S1. Primer sequences used for qRT-PCR analysis genes. 1 NCBI accession number.

Gene	Name	Accession number[1]	Forward Primer (5'-3')	Reverse primer (5'-3')	Annealing temperature (°C)	Amplicon size (bp)
LRP-1	LDL receptor related protein 1	NM_002332.3[1]	GCTATCGACGCCCCTAAGAC	CGCCAGCCCTTTGAGATACA	60	80
RS18	ribosomal protein 18 40S small ribosomal subunit	NM_022551.2[1]	GCAGAATCCACGCCAGTACAA	GCCAGTGGTCTTGGTGTGCT	60	208
RPL32	60S ribosomal protein L32	NM_000994.4[1]	CATTGGTTATGGAAGCAACAAA	TTCTTGGAGGAAACATTGTGAG	60	150
